# RETRACTED: Khan et al. Poly (N-vinylcaprolactam-grafted-sodium alginate) Based Injectable pH/Thermo Responsive In Situ Forming Depot Hydrogels for Prolonged Controlled Anticancer Drug Delivery; In Vitro, In Vivo Characterization and Toxicity Evaluation. *Pharmaceutics* 2022, *14*, 1050

**DOI:** 10.3390/pharmaceutics16010149

**Published:** 2024-01-22

**Authors:** Samiullah Khan, Muhammad Usman Minhas, Muhammad Tahir Aqeel, Ihsan Shah, Shahzeb Khan, Mohsin Kazi, Zachary N. Warnken

**Affiliations:** 1 Margalla College of Pharmacy, Margalla Institute of Health Sciences, Rawalpindi 46000, Punjab, Pakistan; tahiraqeelmalik@gmail.com (M.T.A.); ihsanshah8725@gmail.com (I.S.); 2 College of Pharmacy, University of Sargodha, Sargodha 40100, Punjab, Pakistan; us.minhas@hotmail.com; 3 Department of Pharmacy, University of Malakand, Chakdara 18800, Khyber Pakhtunkhwa, Pakistan; 4 Discipline of Pharmaceutical Sciences, School of Health Sciences, UKZN, Durban 4041, South Africa; 5 Department of Pharmaceutics, College of Pharmacy, King Saud University, P.O. Box 2457, Riyadh 11451, Saudi Arabia; mkazi@ksu.edu.sa; 6 Division of Molecular Pharmaceutics and Drug Delivery, Department of Molecular Pharmaceutics and Drug Delivery, College of Pharmacy, The University of Texas at Austin, Austin, TX 78712, USA; zwarnken@utexas.edu

The journal retracts the article, “Poly (N-vinylcaprolactam-grafted-sodium alginate) Based Injectable pH/Thermo Responsive In Situ Forming Depot Hy-drogels for Prolonged Controlled Anticancer Drug Delivery; In Vitro, In Vivo Characterization and Toxicity Evaluation” [1].

Following publication, concerns were brought to the attention of the publisher regarding overlapping figures with a previously published article [2], with no common authorship and representing different experimental conditions.

Adhering to our complaints procedure, an investigation was conducted by the Editorial Office and the Editorial Board. In this situation, each author was contacted individually to confirm their contribution, and provide original figures. While the authors fully cooperated with the Editorial Office during the investigation, they were unable to satisfactorily explain the overlapping of figures, nor were they able to meet the required quality standards of raw images in order to consider a correction as per the journals original image requirements policy (https://www.mdpi.com/journal/pharmaceutics/instructions#oriimages). As a result, the Editorial Board and Editor-in-Chief were unable to confirm the reliability of the findings and subsequently decided to retract the paper as per MDPI’s retraction policy (https://www.mdpi.com/ethics#_bookmark30).

This retraction was approved by the Editor-in-Chief of *Pharmaceutics*.

The authors disagree with this retraction.
